# 31-Benz­yloxy-5,11,17,23,29-penta-*tert*-butyl­calix[5]arene-32,33,34,35-tetra­ol

**DOI:** 10.1107/S1600536812047435

**Published:** 2012-11-24

**Authors:** Claudia Gargiulli, Giuseppe Gattuso, Anna Notti, Francesco Nicoló, Andrea Pappalardo

**Affiliations:** a Dipartimento di Scienze Chimiche, Università di Messina, Viale F. Stagno d’Alcontres 31, 98166 Messina, Italy; b Dipartimento di Scienze Chimiche, Università di Catania, Viale A. Doria 6, 95125 Catania, Italy

## Abstract

The title compound, C_62_H_76_O_5_, known to be one of the most versatile synthetic precursors/inter­mediates of calix[5]arene derivatives, adopts an approximate *C*_*s*_-symmetric *cone-in* conformation. The aryl­oxybenzyl ring is tilted in such a way that the *p*-*tert*-butyl group fills the macrocycle cavity, while the benzyl group moves away from the cavity axis. In the crystal, this conformational arrangement is secured by intra- and inter­molecular O—H⋯O hydrogen bonds forming inversion dimers. Four *tert*-butyl groups are disordered over two orientations, with occupancy ratios of 0.745 (6):0.255 (6), 0.837 (5):0.163 (5), 0.850 (5):0.150 (5) and 0.845 (8):0.155 (8).

## Related literature

For the synthesis of the title compound, see: Stewart *et al.* (1995 ▶). For calix[5]arene mol­ecules derived from the title compound, see: Garozzo *et al.* (2005 ▶); Capici *et al.* (2011 ▶); Pappalardo *et al.* (2012 ▶). For the structures of calix[5]arene/alkyl­ammonium complexes, see: Gattuso *et al.* (2012 ▶).
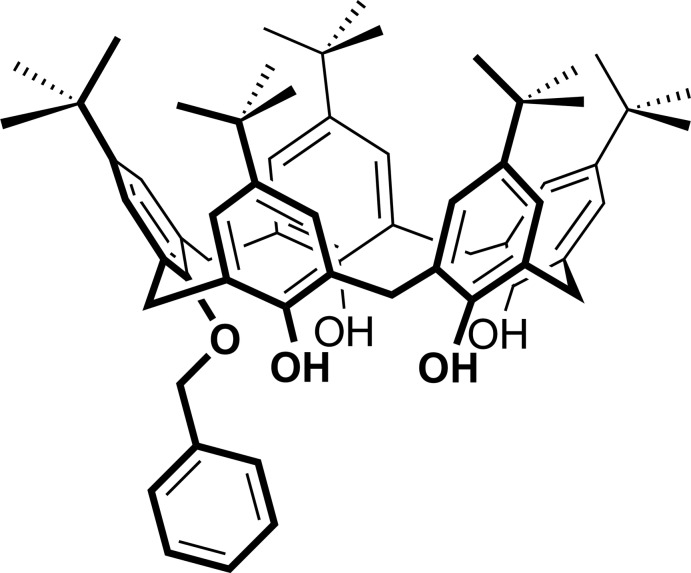


## Experimental

### 

#### Crystal data

C_62_H_76_O_5_
*M* *_r_* = 901.23
Triclinic, 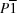

*a* = 14.1467 (8) Å
*b* = 14.3235 (9) Å
*c* = 15.2179 (9) Å
α = 70.653 (3)°
β = 78.776 (3)°
γ = 71.573 (3)°
*V* = 2746.3 (3) Å^3^
*Z* = 2
Mo *K*α radiation
μ = 0.07 mm^−1^
*T* = 296 K
0.38 × 0.18 × 0.10 mm

#### Data collection

Bruker APEXII CCD diffractometer
75089 measured reflections
10532 independent reflections
5343 reflections with *I* > 2σ(*I*)
*R*_int_ = 0.044

#### Refinement

*R*[*F* ^2^ > 2σ(*F* ^2^)] = 0.052
*wR*(*F* ^2^) = 0.161
*S* = 0.96
10532 reflections
677 parameters
120 restraints
H-atom parameters constrained
Δρ_max_ = 0.45 e Å^−3^
Δρ_min_ = −0.16 e Å^−3^

### 

Data collection: *APEX2* (Bruker, 2007 ▶); cell refinement: *SAINT* (Bruker, 2007 ▶); data reduction: *SAINT*; program(s) used to solve structure: *SHELXS97* (Sheldrick, 2008 ▶); program(s) used to refine structure: *SHELXL97* (Sheldrick, 2008 ▶); molecular graphics: *XPW* (Siemens, 1996 ▶); software used to prepare material for publication: *SHELXTL* (Sheldrick, 2008 ▶).

## Supplementary Material

Crystal structure: contains datablock(s) I, global. DOI: 10.1107/S1600536812047435/is5208sup1.cif

Structure factors: contains datablock(s) I. DOI: 10.1107/S1600536812047435/is5208Isup2.hkl

Additional supplementary materials:  crystallographic information; 3D view; checkCIF report

## Figures and Tables

**Table 1:** Hydrogen-bond geometry (Å, °)

*D*—H⋯*A*	*D*—H	H⋯*A*	*D*⋯*A*	*D*—H⋯*A*
O3—H3⋯O5^i^	0.82	2.04	2.7197 (19)	140
O4—H4⋯O3	0.82	2.04	2.847 (2)	168
O5—H5⋯O4	0.82	2.00	2.803 (2)	168

Symmetry code: (i) 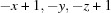.

**Fig. 1.**
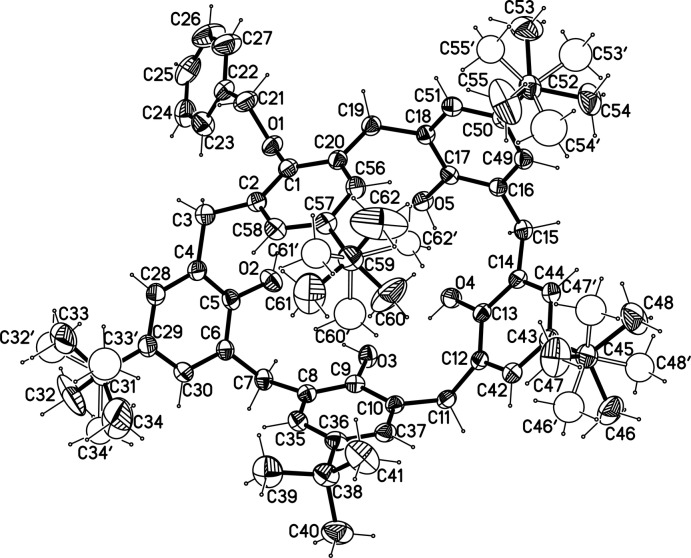
Perspective view of the title molecule with the corresponding atom labelling. Displacement ellipsoids are plotted at a 30% probability level while H atom size is arbitrary.

**Fig. 2.**
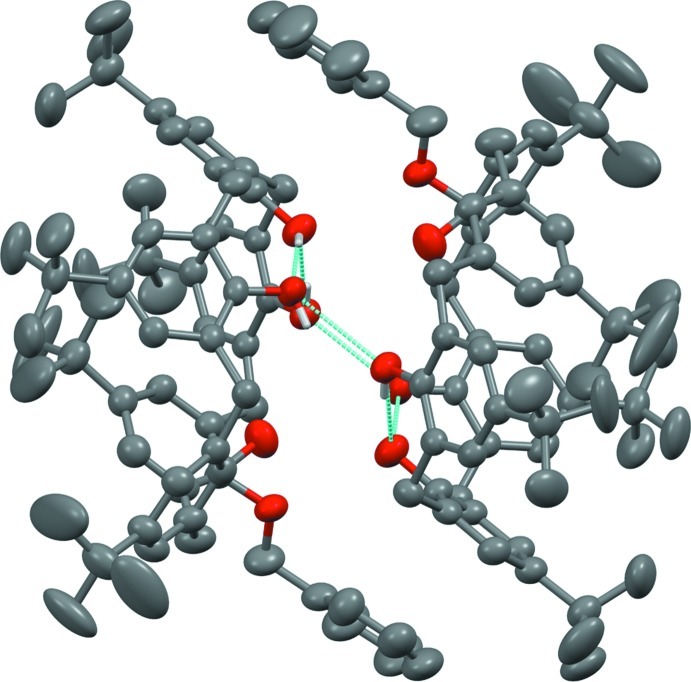
Side view of a centrosymmetric molecular pair (1 - x, -y, 1 - z) showing the sandwiched arrangement of two molecules of the title compound with the hydroxyl group layer located in the middle and the benzyl pendant moieties on the outer surface. Minor components of the disordered tert-butyl groups and H atoms not involved in hydrogen bond interactions have been omitted for clarity. Displacement ellipsoids are plotted at a 50% probability level.

## supplementary crystallographic information

### Comment

In the solid state the title compound adopts an approximate *C*_s_-symmetric *cone-in* conformation, best described by (i) the leaning of the *p*-*tert*-butyl group of the benzylated ring inside the macrocycle cavity (defined by the four remaining *p*-*tert*-butylphenolic rings) and (ii) the concomitant tilting of the benzyl pendant group away from the cavity axis. The crystal packing shows the presence of pairs of centrosymmetric cone-shaped calix[5]arene molecules glued together –via their narrow rims– by several intra- and intermolecular hydrogen-bonding interactions involving the four hydroxyl groups present on each molecule. In addition, the benzyl (C22–C27) moiety and the *p*-*tert*-butylphenolic ring (C12–C14/C44/C43/C42) of each pair of centrosymmetric molecules lie on roughly parallel planes and their edges (C23/C24 and C12/C14, respectively) are seen at a spatial proximity of 3.5 Å (centroid-centroid = 4.47 (2) Å). In analogy with previously reported calixarene structures, collected at low temperature, four *tert*-butyl groups are disordered over two rotated orientations with refined occupancy factors.

### Experimental

The title compound was prepared according to the procedure described by Stewart *et al.* (1995). *p*-*tert*-Butylcalix[5]arene (5.00 g, 6.17 mmol) and anhydrous KHCO_3_ (1.23 g, 12.32 mmol) were suspended in anhydrous MeCN (125 ml). The mixture was heated at reflux for 1 h under an inert atmosphere. Benzyl chloride (0.262 g, 2.09 mmol) was added, and heating was continued for 24 h. The mixture was then allowed to cool, and the solvent was removed under reduced pressure. The resulting residue was partitioned between CHCl_3_ (100 ml) and aqueous HCl (1 *M*, 75 ml). The organic layer was washed with H_2_O (2 × 100 ml) and dried over anhydrous MgSO_4_. The solvent was evaporated under reduced pressure, and the resulting residue was subjected to column chromatography (SiO_2_, toluene/*n*-hexane 1:1 *v*/*v*), to yield the title compound (68%, 1.15 g). Single crystals were obtained by slow evaporation from a CHCl_3_/MeOH solution. Spectral data are in full agreement with those reported in the literature (Stewart *et al.*, 1995).

### Refinement

H atoms were included in the refinement *via* the "riding model" method, with C—H = 0.93–0.97 Å and O—H = 0.82 Å, and with *U*_iso_(H) = 1.2*U*_eq_(C) or 1.5*U*_eq_(O, C_methyl_). Four of the five *tert*-butyl groups showed a rotational disorder. As a result, they were individually treated as an overlap of two rotated conformations and the occupancy factors of the methyl groups were then refined. Owing to the significant disorder, suitable bond and displacement restraints (*DFIX* and *ISOR*) were applied and the C atoms of minor components were refined isotropically.

### Crystal data

**Table:**

C_62_H_76_O_5_	*Z* = 2
*M**_r_* = 901.23	*F*(000) = 976
Triclinic, *P*1	*D*_x_ = 1.090 Mg m^−^^3^
Hall symbol: -P 1	Mo *K*α radiation, λ = 0.71073 Å
*a* = 14.1467 (8) Å	Cell parameters from 9983 reflections
*b* = 14.3235 (9) Å	θ = 2.8–22.1°
*c* = 15.2179 (9) Å	µ = 0.07 mm^−^^1^
α = 70.653 (3)°	*T* = 296 K
β = 78.776 (3)°	Irregular, colourless
γ = 71.573 (3)°	0.38 × 0.18 × 0.10 mm
*V* = 2746.3 (3) Å^3^	

### Data collection

**Table:**

Bruker APEXII CCD diffractometer	5343 reflections with *I* > 2σ(*I*)
Radiation source: fine-focus sealed tube	*R*_int_ = 0.044
Graphite monochromator	θ_max_ = 26.0°, θ_min_ = 2.0°
φ and ω scans	*h* = −17→17
75089 measured reflections	*k* = −17→17
10532 independent reflections	*l* = −18→18

### Refinement

**Table:**

Refinement on *F*^2^	Secondary atom site location: difference Fourier map
Least-squares matrix: full	Hydrogen site location: inferred from neighbouring sites
*R*[*F*^2^ > 2σ(*F*^2^)] = 0.052	H-atom parameters constrained
*wR*(*F*^2^) = 0.161	*w* = 1/[σ^2^(*F*_o_^2^) + (0.0863*P*)^2^] where *P* = (*F*_o_^2^ + 2*F*_c_^2^)/3
*S* = 0.96	(Δ/σ)_max_ = 0.001
10532 reflections	Δρ_max_ = 0.45 e Å^−^^3^
677 parameters	Δρ_min_ = −0.16 e Å^−^^3^
120 restraints	Extinction correction: *SHELXL97* (Sheldrick, 2008), Fc^*^=kFc[1+0.001xFc^2^λ^3^/sin(2θ)]^-1/4^
Primary atom site location: structure-invariant direct methods	Extinction coefficient: 0.0062 (10)

### Special details

**Table:**

Geometry. All s.u.'s (except the s.u. in the dihedral angle between two l.s. planes) are estimated using the full covariance matrix. The cell s.u.'s are taken into account individually in the estimation of s.u.'s in distances, angles and torsion angles; correlations between s.u.'s in cell parameters are only used when they are defined by crystal symmetry. An approximate (isotropic) treatment of cell s.u.'s is used for estimating s.u.'s involving l.s. planes.
Refinement. Refinement of *F*^2^ against ALL reflections. The weighted *R*-factor *wR* and goodness of fit *S* are based on *F*^2^. Conventional *R*-factors *R* are based on *F*, with *F* set to zero for negative *F*^2^. The threshold expression of *F*^2^ > 2σ(*F*^2^) is used only for calculating *R*-factors(gt) *etc*. and is not relevant to the choice of reflections for refinement. *R*-factors based on *F*^2^ are statistically about twice as large as those based on *F*, and *R*- factors based on ALL data will be even larger.

### Fractional atomic coordinates and isotropic or equivalent isotropic displacement parameters (Å^2^)

**Table:**

	*x*	*y*	*z*	*U*_iso_*/*U*_eq_	Occ. (<1)
O1	0.36078 (11)	0.28637 (11)	0.59304 (10)	0.0549 (4)	
O2	0.62075 (12)	0.12493 (12)	0.56199 (12)	0.0650 (5)	
H2	0.5669	0.1678	0.5506	0.098*	
O3	0.71031 (12)	−0.05203 (12)	0.43351 (10)	0.0543 (4)	
H3	0.6972	−0.0509	0.4881	0.081*	
O4	0.55263 (11)	−0.01224 (12)	0.32381 (10)	0.0575 (4)	
H4	0.6024	−0.0311	0.3519	0.086*	
O5	0.37944 (11)	0.11640 (11)	0.39079 (9)	0.0521 (4)	
H5	0.4243	0.0749	0.3693	0.078*	
C1	0.41318 (16)	0.32444 (16)	0.50767 (14)	0.0458 (5)	
C2	0.50301 (17)	0.34459 (17)	0.50671 (15)	0.0520 (6)	
C3	0.54754 (17)	0.3264 (2)	0.59638 (16)	0.0641 (7)	
H3A	0.5417	0.3921	0.6051	0.077*	
H3B	0.5097	0.2896	0.6492	0.077*	
C4	0.65643 (17)	0.26538 (18)	0.59418 (14)	0.0513 (6)	
C5	0.68675 (17)	0.17104 (17)	0.57534 (14)	0.0477 (6)	
C6	0.78683 (16)	0.11664 (16)	0.56911 (14)	0.0448 (5)	
C7	0.81837 (17)	0.01950 (16)	0.54016 (14)	0.0481 (6)	
H7A	0.7693	−0.0191	0.5685	0.058*	
H7B	0.8820	−0.0225	0.5637	0.058*	
C8	0.82882 (15)	0.03977 (15)	0.43458 (14)	0.0415 (5)	
C9	0.78124 (15)	0.00094 (15)	0.38942 (14)	0.0416 (5)	
C10	0.80587 (15)	0.00926 (15)	0.29388 (14)	0.0430 (5)	
C11	0.76412 (16)	−0.04627 (17)	0.24796 (15)	0.0495 (6)	
H11A	0.8178	−0.0770	0.2073	0.059*	
H11B	0.7433	−0.1019	0.2965	0.059*	
C12	0.67691 (16)	0.01793 (16)	0.19121 (15)	0.0455 (5)	
C13	0.57818 (16)	0.02790 (16)	0.22883 (14)	0.0443 (5)	
C14	0.49976 (16)	0.07443 (15)	0.17293 (15)	0.0450 (5)	
C15	0.39230 (16)	0.07875 (16)	0.21118 (15)	0.0488 (6)	
H15A	0.3911	0.0227	0.2683	0.059*	
H15B	0.3592	0.0686	0.1663	0.059*	
C16	0.33380 (15)	0.17948 (15)	0.23208 (14)	0.0412 (5)	
C17	0.33033 (15)	0.19305 (16)	0.31852 (14)	0.0414 (5)	
C18	0.27617 (15)	0.28567 (16)	0.33713 (14)	0.0430 (5)	
C19	0.28254 (16)	0.30445 (17)	0.42731 (14)	0.0482 (6)	
H19A	0.2801	0.2431	0.4788	0.058*	
H19B	0.2253	0.3598	0.4382	0.058*	
C20	0.37805 (16)	0.33231 (15)	0.42491 (14)	0.0448 (5)	
C21	0.2932 (2)	0.3616 (2)	0.63292 (17)	0.0727 (8)	
H21A	0.2359	0.3967	0.5971	0.087*	
H21B	0.3258	0.4122	0.6322	0.087*	
C22	0.25972 (19)	0.3090 (2)	0.73196 (17)	0.0613 (7)	
C23	0.3257 (2)	0.2356 (2)	0.79098 (19)	0.0747 (8)	
H23	0.3933	0.2179	0.7694	0.090*	
C24	0.2951 (3)	0.1873 (2)	0.8811 (2)	0.0867 (9)	
H24	0.3418	0.1378	0.9197	0.104*	
C25	0.1978 (4)	0.2110 (3)	0.9140 (2)	0.1052 (12)	
H25	0.1775	0.1786	0.9754	0.126*	
C26	0.1291 (3)	0.2826 (3)	0.8570 (3)	0.1205 (13)	
H26	0.0614	0.2979	0.8788	0.145*	
C27	0.1609 (2)	0.3329 (3)	0.7654 (2)	0.0993 (10)	
H27	0.1144	0.3831	0.7270	0.119*	
C28	0.73001 (18)	0.30385 (18)	0.60666 (15)	0.0562 (6)	
H28	0.7107	0.3675	0.6180	0.067*	
C29	0.83081 (18)	0.25165 (18)	0.60289 (16)	0.0550 (6)	
C30	0.85644 (17)	0.15751 (17)	0.58484 (14)	0.0514 (6)	
H30	0.9234	0.1201	0.5832	0.062*	
C31	0.91070 (19)	0.29486 (19)	0.61607 (19)	0.0754 (8)	
C32	0.9620 (4)	0.2200 (4)	0.7031 (4)	0.162 (3)	0.745 (6)
H32A	0.9891	0.1527	0.6946	0.243*	0.745 (6)
H32B	0.9138	0.2170	0.7573	0.243*	0.745 (6)
H32C	1.0150	0.2435	0.7118	0.243*	0.745 (6)
C33	0.8703 (3)	0.3968 (3)	0.6352 (5)	0.126 (3)	0.745 (6)
H33A	0.9237	0.4160	0.6489	0.189*	0.745 (6)
H33B	0.8202	0.3922	0.6878	0.189*	0.745 (6)
H33C	0.8411	0.4476	0.5812	0.189*	0.745 (6)
C34	0.9895 (5)	0.2986 (7)	0.5337 (5)	0.200 (5)	0.745 (6)
H34A	1.0450	0.3146	0.5474	0.300*	0.745 (6)
H34B	0.9617	0.3506	0.4796	0.300*	0.745 (6)
H34C	1.0122	0.2330	0.5215	0.300*	0.745 (6)
C32'	0.8858 (11)	0.3258 (13)	0.7054 (7)	0.116 (6)*	0.255 (6)
H32D	0.8904	0.2655	0.7583	0.173*	0.255 (6)
H32E	0.8190	0.3704	0.7070	0.173*	0.255 (6)
H32F	0.9323	0.3611	0.7079	0.173*	0.255 (6)
C33'	0.9007 (11)	0.3965 (8)	0.5359 (8)	0.114 (6)*	0.255 (6)
H33D	0.9500	0.4287	0.5381	0.172*	0.255 (6)
H33E	0.8349	0.4417	0.5430	0.172*	0.255 (6)
H33F	0.9109	0.3823	0.4769	0.172*	0.255 (6)
C34'	1.0182 (5)	0.2400 (10)	0.5963 (9)	0.085 (5)*	0.255 (6)
H34D	1.0284	0.2303	0.5353	0.128*	0.255 (6)
H34E	1.0347	0.1743	0.6425	0.128*	0.255 (6)
H34F	1.0602	0.2802	0.5983	0.128*	0.255 (6)
C35	0.89385 (16)	0.09669 (16)	0.37994 (15)	0.0483 (6)	
H35	0.9242	0.1255	0.4094	0.058*	
C36	0.91619 (16)	0.11311 (17)	0.28425 (15)	0.0502 (6)	
C37	0.87201 (16)	0.06520 (17)	0.24414 (15)	0.0511 (6)	
H37	0.8880	0.0713	0.1806	0.061*	
C38	0.98979 (18)	0.17462 (19)	0.22501 (16)	0.0619 (7)	
C39	1.0147 (2)	0.2362 (2)	0.2786 (2)	0.0927 (9)	
H39A	1.0445	0.1900	0.3341	0.139*	
H39B	0.9545	0.2837	0.2956	0.139*	
H39C	1.0609	0.2734	0.2396	0.139*	
C40	1.0877 (2)	0.1008 (2)	0.1996 (2)	0.0897 (9)	
H40A	1.0751	0.0624	0.1642	0.135*	
H40B	1.1160	0.0544	0.2558	0.135*	
H40C	1.1339	0.1392	0.1629	0.135*	
C41	0.9457 (2)	0.2485 (2)	0.1357 (2)	0.0993 (10)	
H41A	0.9912	0.2883	0.1002	0.149*	
H41B	0.8830	0.2938	0.1514	0.149*	
H41C	0.9352	0.2102	0.0990	0.149*	
C42	0.69514 (17)	0.06404 (17)	0.09611 (15)	0.0522 (6)	
H42	0.7611	0.0605	0.0706	0.063*	
C43	0.61966 (18)	0.11512 (17)	0.03748 (15)	0.0512 (6)	
C44	0.52328 (17)	0.11696 (17)	0.07761 (15)	0.0509 (6)	
H44	0.4714	0.1481	0.0393	0.061*	
C45	0.64528 (17)	0.16541 (18)	−0.06686 (15)	0.0620 (7)	
C46	0.7301 (3)	0.0902 (3)	−0.1089 (2)	0.0960 (15)	0.837 (5)
H46A	0.7137	0.0262	−0.0941	0.144*	0.837 (5)
H46B	0.7912	0.0784	−0.0833	0.144*	0.837 (5)
H46C	0.7387	0.1188	−0.1756	0.144*	0.837 (5)
C47	0.6761 (4)	0.2596 (3)	−0.0760 (2)	0.1068 (16)	0.837 (5)
H47A	0.7349	0.2401	−0.0443	0.160*	0.837 (5)
H47B	0.6228	0.3055	−0.0486	0.160*	0.837 (5)
H47C	0.6905	0.2935	−0.1410	0.160*	0.837 (5)
C48	0.5569 (3)	0.1925 (4)	−0.1228 (2)	0.1080 (17)	0.837 (5)
H48A	0.5764	0.2199	−0.1881	0.162*	0.837 (5)
H48B	0.5022	0.2429	−0.1021	0.162*	0.837 (5)
H48C	0.5363	0.1318	−0.1133	0.162*	0.837 (5)
C46'	0.7528 (6)	0.1730 (16)	−0.0891 (14)	0.102 (7)*	0.163 (5)
H46D	0.7610	0.2196	−0.0600	0.153*	0.163 (5)
H46E	0.7676	0.1978	−0.1557	0.153*	0.163 (5)
H46F	0.7977	0.1062	−0.0658	0.153*	0.163 (5)
C47'	0.5730 (12)	0.2719 (8)	−0.0950 (13)	0.099 (7)*	0.163 (5)
H47D	0.5847	0.3026	−0.1611	0.149*	0.163 (5)
H47E	0.5832	0.3139	−0.0618	0.149*	0.163 (5)
H47F	0.5054	0.2665	−0.0798	0.149*	0.163 (5)
C48'	0.6344 (15)	0.0990 (12)	−0.1214 (12)	0.094 (7)*	0.163 (5)
H48D	0.6556	0.1261	−0.1864	0.141*	0.163 (5)
H48E	0.5656	0.0986	−0.1145	0.141*	0.163 (5)
H48F	0.6752	0.0301	−0.0979	0.141*	0.163 (5)
C49	0.28559 (15)	0.26181 (17)	0.16264 (14)	0.0464 (5)	
H49	0.2892	0.2532	0.1040	0.056*	
C50	0.23254 (16)	0.35584 (17)	0.17608 (15)	0.0467 (5)	
C51	0.22843 (16)	0.36418 (17)	0.26557 (15)	0.0487 (6)	
H51	0.1916	0.4259	0.2776	0.058*	
C52	0.18700 (18)	0.44912 (17)	0.09701 (15)	0.0588 (6)	
C53	0.0794 (3)	0.4987 (3)	0.1274 (3)	0.1048 (16)	0.850 (5)
H53A	0.0526	0.5586	0.0781	0.157*	0.850 (5)
H53B	0.0769	0.5181	0.1826	0.157*	0.850 (5)
H53C	0.0404	0.4507	0.1407	0.157*	0.850 (5)
C54	0.1829 (3)	0.4194 (2)	0.0103 (2)	0.0907 (14)	0.850 (5)
H54A	0.1498	0.3658	0.0281	0.136*	0.850 (5)
H54B	0.2497	0.3954	−0.0170	0.136*	0.850 (5)
H54C	0.1465	0.4783	−0.0344	0.136*	0.850 (5)
C55	0.2503 (4)	0.5231 (3)	0.0704 (3)	0.125 (2)	0.850 (5)
H55A	0.2262	0.5792	0.0171	0.187*	0.850 (5)
H55B	0.3186	0.4883	0.0551	0.187*	0.850 (5)
H55C	0.2465	0.5491	0.1220	0.187*	0.850 (5)
C53'	0.0982 (12)	0.4379 (19)	0.0641 (17)	0.122 (10)*	0.150 (5)
H53D	0.0717	0.4989	0.0156	0.183*	0.150 (5)
H53E	0.0475	0.4281	0.1157	0.183*	0.150 (5)
H53F	0.1190	0.3796	0.0402	0.183*	0.150 (5)
C54'	0.2703 (14)	0.462 (2)	0.0183 (14)	0.142 (11)*	0.150 (5)
H54D	0.3029	0.3975	0.0057	0.212*	0.150 (5)
H54E	0.3181	0.4857	0.0363	0.212*	0.150 (5)
H54F	0.2427	0.5118	−0.0370	0.212*	0.150 (5)
C55'	0.1526 (15)	0.5447 (10)	0.1309 (13)	0.084 (7)*	0.150 (5)
H55D	0.1199	0.6025	0.0828	0.126*	0.150 (5)
H55E	0.2096	0.5581	0.1448	0.126*	0.150 (5)
H55F	0.1066	0.5337	0.1862	0.126*	0.150 (5)
C56	0.43687 (17)	0.36095 (16)	0.34192 (15)	0.0508 (6)	
H56	0.4143	0.3671	0.2863	0.061*	
C57	0.52754 (17)	0.38087 (17)	0.33753 (15)	0.0519 (6)	
C58	0.55820 (18)	0.37356 (18)	0.42081 (17)	0.0573 (6)	
H58	0.6179	0.3885	0.4195	0.069*	
C59	0.59229 (18)	0.40210 (19)	0.24439 (16)	0.0663 (7)	
C60	0.6308 (5)	0.3040 (3)	0.2156 (4)	0.134 (2)	0.845 (8)
H60A	0.6693	0.3167	0.1556	0.202*	0.845 (8)
H60B	0.5752	0.2812	0.2117	0.202*	0.845 (8)
H60C	0.6723	0.2518	0.2612	0.202*	0.845 (8)
C61	0.6830 (4)	0.4321 (6)	0.2516 (3)	0.145 (3)	0.845 (8)
H61A	0.7216	0.4445	0.1916	0.218*	0.845 (8)
H61B	0.7233	0.3775	0.2966	0.218*	0.845 (8)
H61C	0.6618	0.4935	0.2710	0.218*	0.845 (8)
C62	0.5313 (3)	0.4880 (6)	0.1718 (3)	0.189 (4)	0.845 (8)
H62A	0.5049	0.5480	0.1933	0.284*	0.845 (8)
H62B	0.4771	0.4670	0.1618	0.284*	0.845 (8)
H62C	0.5731	0.5035	0.1141	0.284*	0.845 (8)
C60'	0.6857 (12)	0.3130 (14)	0.2529 (16)	0.113 (10)*	0.155 (8)
H60D	0.7162	0.3093	0.1916	0.169*	0.155 (8)
H60E	0.6683	0.2502	0.2873	0.169*	0.155 (8)
H60F	0.7320	0.3229	0.2853	0.169*	0.155 (8)
C61'	0.6164 (19)	0.5021 (11)	0.2300 (15)	0.091 (8)*	0.155 (8)
H61D	0.6569	0.4936	0.2774	0.136*	0.155 (8)
H61E	0.5553	0.5550	0.2342	0.136*	0.155 (8)
H61F	0.6522	0.5212	0.1694	0.136*	0.155 (8)
C62'	0.5416 (13)	0.4107 (16)	0.1617 (9)	0.065 (6)*	0.155 (8)
H62D	0.4793	0.4634	0.1586	0.097*	0.155 (8)
H62E	0.5293	0.3462	0.1692	0.097*	0.155 (8)
H62F	0.5843	0.4281	0.1049	0.097*	0.155 (8)

### Atomic displacement parameters (Å^2^)

**Table:**

	*U*^11^	*U*^22^	*U*^33^	*U*^12^	*U*^13^	*U*^23^
O1	0.0611 (10)	0.0554 (10)	0.0425 (9)	−0.0073 (8)	0.0004 (7)	−0.0183 (7)
O2	0.0566 (11)	0.0627 (11)	0.0809 (12)	−0.0222 (8)	−0.0185 (9)	−0.0150 (9)
O3	0.0653 (11)	0.0633 (10)	0.0404 (9)	−0.0316 (8)	−0.0003 (8)	−0.0124 (8)
O4	0.0513 (10)	0.0703 (11)	0.0436 (9)	−0.0118 (8)	−0.0040 (7)	−0.0119 (8)
O5	0.0561 (10)	0.0479 (9)	0.0418 (9)	−0.0060 (7)	−0.0028 (7)	−0.0086 (7)
C1	0.0486 (14)	0.0435 (13)	0.0394 (13)	−0.0040 (10)	−0.0044 (10)	−0.0126 (10)
C2	0.0489 (14)	0.0572 (14)	0.0487 (15)	−0.0046 (11)	−0.0097 (11)	−0.0205 (11)
C3	0.0549 (16)	0.0833 (18)	0.0576 (16)	−0.0056 (13)	−0.0125 (12)	−0.0336 (14)
C4	0.0521 (15)	0.0582 (15)	0.0427 (13)	−0.0078 (12)	−0.0096 (10)	−0.0176 (11)
C5	0.0495 (14)	0.0564 (15)	0.0392 (13)	−0.0180 (12)	−0.0099 (10)	−0.0099 (11)
C6	0.0515 (14)	0.0464 (13)	0.0366 (12)	−0.0138 (11)	−0.0098 (10)	−0.0083 (10)
C7	0.0578 (14)	0.0451 (13)	0.0407 (13)	−0.0147 (11)	−0.0118 (10)	−0.0071 (10)
C8	0.0425 (12)	0.0370 (12)	0.0406 (12)	−0.0075 (10)	−0.0058 (10)	−0.0078 (10)
C9	0.0408 (12)	0.0368 (12)	0.0411 (13)	−0.0097 (10)	−0.0027 (10)	−0.0053 (10)
C10	0.0418 (13)	0.0425 (13)	0.0406 (13)	−0.0052 (10)	−0.0044 (10)	−0.0126 (10)
C11	0.0483 (14)	0.0520 (14)	0.0460 (13)	−0.0054 (11)	−0.0017 (10)	−0.0209 (11)
C12	0.0482 (14)	0.0471 (13)	0.0429 (13)	−0.0073 (10)	−0.0064 (10)	−0.0194 (11)
C13	0.0496 (14)	0.0447 (13)	0.0363 (13)	−0.0090 (10)	−0.0014 (10)	−0.0139 (10)
C14	0.0460 (13)	0.0434 (13)	0.0484 (14)	−0.0095 (10)	−0.0037 (11)	−0.0200 (11)
C15	0.0499 (14)	0.0511 (14)	0.0505 (13)	−0.0159 (11)	−0.0070 (10)	−0.0182 (11)
C16	0.0381 (12)	0.0472 (13)	0.0406 (13)	−0.0158 (10)	−0.0006 (9)	−0.0136 (11)
C17	0.0388 (12)	0.0452 (13)	0.0373 (13)	−0.0141 (10)	−0.0053 (9)	−0.0048 (10)
C18	0.0440 (13)	0.0460 (13)	0.0387 (12)	−0.0147 (10)	−0.0020 (10)	−0.0108 (10)
C19	0.0515 (14)	0.0486 (13)	0.0428 (13)	−0.0111 (11)	−0.0036 (10)	−0.0140 (10)
C20	0.0489 (14)	0.0409 (13)	0.0423 (13)	−0.0070 (10)	−0.0073 (10)	−0.0124 (10)
C21	0.0689 (18)	0.0747 (18)	0.0664 (18)	−0.0004 (14)	0.0017 (14)	−0.0332 (15)
C22	0.0600 (17)	0.0704 (17)	0.0572 (16)	−0.0131 (14)	0.0031 (13)	−0.0329 (14)
C23	0.0705 (19)	0.093 (2)	0.0608 (18)	−0.0297 (16)	−0.0067 (15)	−0.0147 (16)
C24	0.105 (3)	0.103 (2)	0.064 (2)	−0.044 (2)	−0.0059 (18)	−0.0268 (18)
C25	0.144 (4)	0.109 (3)	0.067 (2)	−0.043 (3)	0.026 (2)	−0.043 (2)
C26	0.100 (3)	0.134 (3)	0.102 (3)	−0.012 (3)	0.042 (2)	−0.049 (3)
C27	0.081 (2)	0.108 (2)	0.087 (2)	0.0032 (19)	0.0102 (18)	−0.038 (2)
C28	0.0598 (16)	0.0561 (15)	0.0588 (15)	−0.0097 (12)	−0.0154 (12)	−0.0247 (12)
C29	0.0571 (16)	0.0549 (15)	0.0587 (15)	−0.0120 (12)	−0.0155 (12)	−0.0214 (12)
C30	0.0473 (14)	0.0562 (15)	0.0513 (14)	−0.0101 (11)	−0.0111 (11)	−0.0168 (12)
C31	0.0654 (18)	0.0744 (19)	0.104 (2)	−0.0210 (15)	−0.0191 (16)	−0.0402 (17)
C32	0.151 (6)	0.123 (5)	0.249 (8)	−0.036 (4)	−0.144 (6)	−0.032 (4)
C33	0.100 (4)	0.087 (4)	0.234 (7)	−0.020 (3)	−0.052 (4)	−0.088 (4)
C34	0.172 (7)	0.322 (12)	0.228 (9)	−0.194 (8)	0.091 (6)	−0.182 (9)
C35	0.0466 (14)	0.0514 (14)	0.0469 (14)	−0.0146 (11)	−0.0096 (10)	−0.0104 (11)
C36	0.0424 (13)	0.0524 (14)	0.0485 (14)	−0.0123 (11)	−0.0027 (10)	−0.0070 (11)
C37	0.0452 (14)	0.0609 (15)	0.0382 (13)	−0.0087 (12)	0.0001 (10)	−0.0107 (11)
C38	0.0568 (16)	0.0661 (16)	0.0555 (15)	−0.0265 (13)	0.0014 (12)	−0.0027 (13)
C39	0.102 (2)	0.096 (2)	0.089 (2)	−0.0631 (19)	0.0056 (17)	−0.0130 (18)
C40	0.0609 (19)	0.114 (2)	0.082 (2)	−0.0322 (17)	0.0139 (15)	−0.0171 (18)
C41	0.098 (2)	0.105 (2)	0.078 (2)	−0.0536 (19)	−0.0187 (17)	0.0263 (18)
C42	0.0497 (14)	0.0626 (15)	0.0461 (14)	−0.0143 (12)	−0.0014 (11)	−0.0208 (12)
C43	0.0562 (16)	0.0551 (14)	0.0434 (13)	−0.0122 (11)	−0.0039 (11)	−0.0192 (11)
C44	0.0512 (15)	0.0573 (14)	0.0446 (14)	−0.0092 (11)	−0.0099 (11)	−0.0176 (11)
C45	0.0644 (17)	0.0718 (17)	0.0448 (14)	−0.0175 (13)	−0.0060 (12)	−0.0113 (13)
C46	0.105 (3)	0.123 (3)	0.052 (2)	−0.021 (2)	0.0083 (19)	−0.033 (2)
C47	0.162 (5)	0.093 (3)	0.066 (2)	−0.068 (3)	−0.006 (2)	0.005 (2)
C48	0.091 (3)	0.173 (5)	0.046 (2)	−0.043 (3)	−0.0165 (18)	−0.001 (2)
C49	0.0450 (13)	0.0565 (15)	0.0404 (13)	−0.0173 (11)	−0.0035 (10)	−0.0144 (11)
C50	0.0468 (13)	0.0493 (14)	0.0434 (13)	−0.0146 (11)	−0.0076 (10)	−0.0094 (11)
C51	0.0472 (14)	0.0454 (13)	0.0511 (14)	−0.0087 (10)	−0.0066 (11)	−0.0138 (11)
C52	0.0687 (17)	0.0534 (15)	0.0483 (14)	−0.0135 (13)	−0.0164 (12)	−0.0038 (12)
C53	0.095 (3)	0.089 (3)	0.089 (3)	0.025 (2)	−0.030 (2)	−0.008 (2)
C54	0.126 (4)	0.075 (2)	0.063 (2)	−0.010 (2)	−0.048 (2)	−0.0056 (18)
C55	0.180 (5)	0.095 (3)	0.105 (3)	−0.092 (3)	−0.070 (3)	0.046 (3)
C56	0.0562 (15)	0.0547 (14)	0.0409 (13)	−0.0146 (11)	−0.0096 (11)	−0.0108 (11)
C57	0.0550 (15)	0.0488 (14)	0.0487 (14)	−0.0138 (11)	−0.0046 (11)	−0.0106 (11)
C58	0.0504 (15)	0.0621 (16)	0.0617 (16)	−0.0155 (12)	−0.0092 (12)	−0.0189 (12)
C59	0.0623 (17)	0.0794 (19)	0.0546 (16)	−0.0277 (14)	0.0027 (13)	−0.0126 (14)
C60	0.148 (5)	0.153 (4)	0.127 (4)	−0.080 (4)	0.081 (4)	−0.089 (3)
C61	0.138 (5)	0.248 (8)	0.103 (3)	−0.143 (6)	0.052 (3)	−0.071 (4)
C62	0.122 (4)	0.198 (7)	0.096 (4)	0.010 (4)	0.027 (3)	0.081 (4)

### Geometric parameters (Å, º)

**Table:**

O1—C1	1.395 (2)	C38—C40	1.524 (4)
O1—C21	1.414 (3)	C38—C39	1.534 (4)
O2—C5	1.379 (3)	C39—H39A	0.9600
O2—H2	0.8200	C39—H39B	0.9600
O3—C9	1.384 (2)	C39—H39C	0.9600
O3—H3	0.8200	C40—H40A	0.9600
O4—C13	1.388 (2)	C40—H40B	0.9600
O4—H4	0.8200	C40—H40C	0.9600
O5—C17	1.383 (2)	C41—H41A	0.9600
O5—H5	0.8200	C41—H41B	0.9600
C1—C2	1.386 (3)	C41—H41C	0.9600
C1—C20	1.400 (3)	C42—C43	1.384 (3)
C2—C58	1.392 (3)	C42—H42	0.9300
C2—C3	1.524 (3)	C43—C44	1.377 (3)
C3—C4	1.512 (3)	C43—C45	1.534 (3)
C3—H3A	0.9700	C44—H44	0.9300
C3—H3B	0.9700	C45—C47	1.502 (3)
C4—C28	1.390 (3)	C45—C48'	1.511 (5)
C4—C5	1.391 (3)	C45—C47'	1.519 (5)
C5—C6	1.386 (3)	C45—C48	1.526 (3)
C6—C30	1.385 (3)	C45—C46'	1.523 (5)
C6—C7	1.505 (3)	C45—C46	1.533 (3)
C7—C8	1.521 (3)	C46—H46A	0.9600
C7—H7A	0.9700	C46—H46B	0.9600
C7—H7B	0.9700	C46—H46C	0.9600
C8—C9	1.380 (3)	C47—H47A	0.9600
C8—C35	1.389 (3)	C47—H47B	0.9600
C9—C10	1.401 (3)	C47—H47C	0.9600
C10—C37	1.374 (3)	C48—H48A	0.9600
C10—C11	1.515 (3)	C48—H48B	0.9600
C11—C12	1.512 (3)	C48—H48C	0.9600
C11—H11A	0.9700	C46'—H46D	0.9600
C11—H11B	0.9700	C46'—H46E	0.9600
C12—C13	1.384 (3)	C46'—H46F	0.9600
C12—C42	1.388 (3)	C47'—H47D	0.9600
C13—C14	1.387 (3)	C47'—H47E	0.9600
C14—C44	1.393 (3)	C47'—H47F	0.9600
C14—C15	1.508 (3)	C48'—H48D	0.9600
C15—C16	1.521 (3)	C48'—H48E	0.9600
C15—H15A	0.9700	C48'—H48F	0.9600
C15—H15B	0.9700	C49—C50	1.379 (3)
C16—C17	1.382 (3)	C49—H49	0.9300
C16—C49	1.385 (3)	C50—C51	1.395 (3)
C17—C18	1.396 (3)	C50—C52	1.530 (3)
C18—C51	1.379 (3)	C51—H51	0.9300
C18—C19	1.507 (3)	C52—C55	1.505 (3)
C19—C20	1.516 (3)	C52—C53'	1.510 (5)
C19—H19A	0.9700	C52—C53	1.515 (3)
C19—H19B	0.9700	C52—C54'	1.517 (5)
C20—C56	1.385 (3)	C52—C55'	1.525 (5)
C21—C22	1.503 (3)	C52—C54	1.531 (3)
C21—H21A	0.9700	C53—H53A	0.9600
C21—H21B	0.9700	C53—H53B	0.9600
C22—C27	1.366 (4)	C53—H53C	0.9600
C22—C23	1.365 (3)	C54—H54A	0.9600
C23—C24	1.369 (4)	C54—H54B	0.9600
C23—H23	0.9300	C54—H54C	0.9600
C24—C25	1.345 (4)	C55—H55A	0.9600
C24—H24	0.9300	C55—H55B	0.9600
C25—C26	1.363 (5)	C55—H55C	0.9600
C25—H25	0.9300	C53'—H53D	0.9600
C26—C27	1.400 (4)	C53'—H53E	0.9600
C26—H26	0.9300	C53'—H53F	0.9600
C27—H27	0.9300	C54'—H54D	0.9600
C28—C29	1.385 (3)	C54'—H54E	0.9600
C28—H28	0.9300	C54'—H54F	0.9600
C29—C30	1.387 (3)	C55'—H55D	0.9600
C29—C31	1.521 (3)	C55'—H55E	0.9600
C30—H30	0.9300	C55'—H55F	0.9600
C31—C34'	1.498 (5)	C56—C57	1.384 (3)
C31—C33	1.494 (4)	C56—H56	0.9300
C31—C34	1.506 (4)	C57—C58	1.379 (3)
C31—C32'	1.511 (5)	C57—C59	1.525 (3)
C31—C32	1.541 (4)	C58—H58	0.9300
C31—C33'	1.545 (5)	C59—C61'	1.513 (5)
C32—H32A	0.9600	C59—C60	1.515 (3)
C32—H32B	0.9600	C59—C62	1.509 (4)
C32—H32C	0.9600	C59—C60'	1.511 (5)
C33—H33A	0.9600	C59—C61	1.510 (4)
C33—H33B	0.9600	C59—C62'	1.518 (5)
C33—H33C	0.9600	C60—H60A	0.9600
C34—H34A	0.9600	C60—H60B	0.9600
C34—H34B	0.9600	C60—H60C	0.9600
C34—H34C	0.9600	C61—H61A	0.9600
C32'—H32D	0.9600	C61—H61B	0.9600
C32'—H32E	0.9600	C61—H61C	0.9600
C32'—H32F	0.9600	C62—H62A	0.9600
C33'—H33D	0.9600	C62—H62B	0.9600
C33'—H33E	0.9600	C62—H62C	0.9600
C33'—H33F	0.9600	C60'—H60D	0.9600
C34'—H34D	0.9600	C60'—H60E	0.9600
C34'—H34E	0.9600	C60'—H60F	0.9600
C34'—H34F	0.9600	C61'—H61D	0.9600
C35—C36	1.384 (3)	C61'—H61E	0.9600
C35—H35	0.9300	C61'—H61F	0.9600
C36—C37	1.390 (3)	C62'—H62D	0.9600
C36—C38	1.537 (3)	C62'—H62E	0.9600
C37—H37	0.9300	C62'—H62F	0.9600
C38—C41	1.522 (3)		
			
C1—O1—C21	115.40 (17)	C39—C38—C36	111.8 (2)
C5—O2—H2	109.5	C38—C39—H39A	109.5
C9—O3—H3	109.5	C38—C39—H39B	109.5
C13—O4—H4	109.5	H39A—C39—H39B	109.5
C17—O5—H5	109.5	C38—C39—H39C	109.5
C2—C1—O1	119.59 (19)	H39A—C39—H39C	109.5
C2—C1—C20	121.4 (2)	H39B—C39—H39C	109.5
O1—C1—C20	118.7 (2)	C38—C40—H40A	109.5
C1—C2—C58	118.4 (2)	C38—C40—H40B	109.5
C1—C2—C3	122.2 (2)	H40A—C40—H40B	109.5
C58—C2—C3	119.2 (2)	C38—C40—H40C	109.5
C4—C3—C2	112.05 (18)	H40A—C40—H40C	109.5
C4—C3—H3A	109.2	H40B—C40—H40C	109.5
C2—C3—H3A	109.2	C38—C41—H41A	109.5
C4—C3—H3B	109.2	C38—C41—H41B	109.5
C2—C3—H3B	109.2	H41A—C41—H41B	109.5
H3A—C3—H3B	107.9	C38—C41—H41C	109.5
C28—C4—C5	117.8 (2)	H41A—C41—H41C	109.5
C28—C4—C3	121.1 (2)	H41B—C41—H41C	109.5
C5—C4—C3	121.0 (2)	C43—C42—C12	123.0 (2)
O2—C5—C6	115.4 (2)	C43—C42—H42	118.5
O2—C5—C4	123.1 (2)	C12—C42—H42	118.5
C6—C5—C4	121.5 (2)	C44—C43—C42	116.7 (2)
C30—C6—C5	118.0 (2)	C44—C43—C45	123.2 (2)
C30—C6—C7	121.5 (2)	C42—C43—C45	120.1 (2)
C5—C6—C7	120.31 (19)	C43—C44—C14	123.1 (2)
C6—C7—C8	112.92 (16)	C43—C44—H44	118.4
C6—C7—H7A	109.0	C14—C44—H44	118.4
C8—C7—H7A	109.0	C48'—C45—C47'	110.7 (9)
C6—C7—H7B	109.0	C47—C45—C48	111.0 (3)
C8—C7—H7B	109.0	C48'—C45—C46'	107.1 (9)
H7A—C7—H7B	107.8	C47'—C45—C46'	110.1 (9)
C9—C8—C35	117.42 (19)	C48—C45—C46'	136.0 (8)
C9—C8—C7	124.07 (19)	C47—C45—C43	108.1 (2)
C35—C8—C7	118.43 (19)	C48'—C45—C43	108.2 (7)
C8—C9—O3	124.07 (18)	C47'—C45—C43	108.3 (8)
C8—C9—C10	121.02 (19)	C48—C45—C43	111.5 (2)
O3—C9—C10	114.86 (18)	C46'—C45—C43	112.5 (8)
C37—C10—C9	118.3 (2)	C47—C45—C46	110.4 (3)
C37—C10—C11	121.01 (19)	C48—C45—C46	105.5 (3)
C9—C10—C11	120.61 (19)	C43—C45—C46	110.5 (2)
C12—C11—C10	116.47 (17)	C45—C46—H46A	109.5
C12—C11—H11A	108.2	C45—C46—H46B	109.5
C10—C11—H11A	108.2	C45—C46—H46C	109.5
C12—C11—H11B	108.2	C45—C47—H47A	109.5
C10—C11—H11B	108.2	C45—C47—H47B	109.5
H11A—C11—H11B	107.3	C45—C47—H47C	109.5
C13—C12—C42	117.8 (2)	C45—C48—H48A	109.5
C13—C12—C11	122.67 (19)	C45—C48—H48B	109.5
C42—C12—C11	119.4 (2)	C45—C48—H48C	109.5
C12—C13—C14	121.65 (19)	C45—C46'—H46D	109.5
C12—C13—O4	121.84 (19)	C45—C46'—H46E	109.5
C14—C13—O4	116.45 (19)	H46D—C46'—H46E	109.5
C13—C14—C44	117.6 (2)	C45—C46'—H46F	109.5
C13—C14—C15	122.64 (19)	H46D—C46'—H46F	109.5
C44—C14—C15	119.80 (19)	H46E—C46'—H46F	109.5
C14—C15—C16	113.64 (17)	C45—C47'—H47D	109.5
C14—C15—H15A	108.8	C45—C47'—H47E	109.5
C16—C15—H15A	108.8	H47D—C47'—H47E	109.5
C14—C15—H15B	108.8	C45—C47'—H47F	109.5
C16—C15—H15B	108.8	H47D—C47'—H47F	109.5
H15A—C15—H15B	107.7	H47E—C47'—H47F	109.5
C17—C16—C49	118.26 (19)	C45—C48'—H48D	109.5
C17—C16—C15	121.58 (18)	C45—C48'—H48E	109.5
C49—C16—C15	120.11 (18)	H48D—C48'—H48E	109.5
C16—C17—O5	122.21 (19)	C45—C48'—H48F	109.5
C16—C17—C18	121.16 (18)	H48D—C48'—H48F	109.5
O5—C17—C18	116.63 (18)	H48E—C48'—H48F	109.5
C51—C18—C17	117.78 (19)	C50—C49—C16	123.32 (19)
C51—C18—C19	120.78 (19)	C50—C49—H49	118.3
C17—C18—C19	120.81 (18)	C16—C49—H49	118.3
C18—C19—C20	111.67 (17)	C49—C50—C51	116.04 (19)
C18—C19—H19A	109.3	C49—C50—C52	123.01 (19)
C20—C19—H19A	109.3	C51—C50—C52	120.8 (2)
C18—C19—H19B	109.3	C18—C51—C50	123.4 (2)
C20—C19—H19B	109.3	C18—C51—H51	118.3
H19A—C19—H19B	107.9	C50—C51—H51	118.3
C56—C20—C1	117.3 (2)	C55—C52—C53	111.6 (3)
C56—C20—C19	121.72 (19)	C53'—C52—C54'	111.4 (11)
C1—C20—C19	120.92 (19)	C55—C52—C50	108.4 (2)
O1—C21—C22	108.4 (2)	C53'—C52—C50	112.9 (10)
O1—C21—H21A	110.0	C53—C52—C50	110.3 (2)
C22—C21—H21A	110.0	C54'—C52—C50	106.6 (11)
O1—C21—H21B	110.0	C53'—C52—C55'	108.2 (10)
C22—C21—H21B	110.0	C54'—C52—C55'	108.5 (10)
H21A—C21—H21B	108.4	C50—C52—C55'	109.3 (7)
C27—C22—C23	117.8 (3)	C55—C52—C54	108.8 (3)
C27—C22—C21	120.5 (3)	C53—C52—C54	105.5 (2)
C23—C22—C21	121.8 (2)	C50—C52—C54	112.1 (2)
C22—C23—C24	121.7 (3)	C55'—C52—C54	138.4 (7)
C22—C23—H23	119.1	C52—C53—H53A	109.5
C24—C23—H23	119.1	C52—C53—H53B	109.5
C25—C24—C23	120.4 (3)	C52—C53—H53C	109.5
C25—C24—H24	119.8	C52—C54—H54A	109.5
C23—C24—H24	119.8	C52—C54—H54B	109.5
C24—C25—C26	119.8 (3)	C52—C54—H54C	109.5
C24—C25—H25	120.1	C52—C55—H55A	109.5
C26—C25—H25	120.1	C52—C55—H55B	109.5
C25—C26—C27	119.5 (3)	C52—C55—H55C	109.5
C25—C26—H26	120.2	C52—C53'—H53D	109.5
C27—C26—H26	120.2	C52—C53'—H53E	109.5
C22—C27—C26	120.7 (3)	H53D—C53'—H53E	109.5
C22—C27—H27	119.7	C52—C53'—H53F	109.5
C26—C27—H27	119.7	H53D—C53'—H53F	109.5
C29—C28—C4	123.0 (2)	H53E—C53'—H53F	109.5
C29—C28—H28	118.5	C52—C54'—H54D	109.5
C4—C28—H28	118.5	C52—C54'—H54E	109.5
C30—C29—C28	116.6 (2)	H54D—C54'—H54E	109.5
C30—C29—C31	120.9 (2)	C52—C54'—H54F	109.5
C28—C29—C31	122.5 (2)	H54D—C54'—H54F	109.5
C29—C30—C6	123.0 (2)	H54E—C54'—H54F	109.5
C29—C30—H30	118.5	C52—C55'—H55D	109.5
C6—C30—H30	118.5	C52—C55'—H55E	109.5
C33—C31—C34	111.3 (4)	H55D—C55'—H55E	109.5
C34'—C31—C32'	115.3 (7)	C52—C55'—H55F	109.5
C34'—C31—C29	117.9 (6)	H55D—C55'—H55F	109.5
C33—C31—C29	114.0 (3)	H55E—C55'—H55F	109.5
C34—C31—C29	110.3 (3)	C57—C56—C20	123.4 (2)
C32'—C31—C29	111.2 (6)	C57—C56—H56	118.3
C33—C31—C32	106.0 (3)	C20—C56—H56	118.3
C34—C31—C32	107.5 (5)	C58—C57—C56	117.2 (2)
C29—C31—C32	107.4 (2)	C58—C57—C59	122.7 (2)
C34'—C31—C33'	100.9 (7)	C56—C57—C59	120.0 (2)
C32'—C31—C33'	105.5 (8)	C57—C58—C2	122.4 (2)
C29—C31—C33'	103.8 (6)	C57—C58—H58	118.8
C31—C32—H32A	109.5	C2—C58—H58	118.8
C31—C32—H32B	109.5	C60—C59—C62	111.2 (4)
C31—C32—H32C	109.5	C61'—C59—C60'	111.6 (10)
C31—C33—H33A	109.5	C61'—C59—C57	107.1 (9)
C31—C33—H33B	109.5	C60—C59—C57	108.3 (2)
C31—C33—H33C	109.5	C62—C59—C57	109.7 (2)
C31—C34—H34A	109.5	C60'—C59—C57	106.8 (9)
C31—C34—H34B	109.5	C60—C59—C61	106.8 (3)
C31—C34—H34C	109.5	C62—C59—C61	108.6 (4)
C31—C32'—H32D	109.5	C57—C59—C61	112.3 (2)
C31—C32'—H32E	109.5	C61'—C59—C62'	109.4 (9)
H32D—C32'—H32E	109.5	C60'—C59—C62'	108.6 (9)
C31—C32'—H32F	109.5	C57—C59—C62'	113.4 (7)
H32D—C32'—H32F	109.5	C59—C60—H60A	109.5
H32E—C32'—H32F	109.5	C59—C60—H60B	109.5
C31—C33'—H33D	109.5	C59—C60—H60C	109.5
C31—C33'—H33E	109.5	C59—C61—H61A	109.5
H33D—C33'—H33E	109.5	C59—C61—H61B	109.5
C31—C33'—H33F	109.5	C59—C61—H61C	109.5
H33D—C33'—H33F	109.5	C59—C62—H62A	109.5
H33E—C33'—H33F	109.5	C59—C62—H62B	109.5
C31—C34'—H34D	109.5	C59—C62—H62C	109.5
C31—C34'—H34E	109.5	C59—C60'—H60D	109.5
H34D—C34'—H34E	109.5	C59—C60'—H60E	109.5
C31—C34'—H34F	109.5	H60D—C60'—H60E	109.5
H34D—C34'—H34F	109.5	C59—C60'—H60F	109.5
H34E—C34'—H34F	109.5	H60D—C60'—H60F	109.5
C36—C35—C8	123.9 (2)	H60E—C60'—H60F	109.5
C36—C35—H35	118.0	C59—C61'—H61D	109.5
C8—C35—H35	118.0	C59—C61'—H61E	109.5
C35—C36—C37	115.8 (2)	H61D—C61'—H61E	109.5
C35—C36—C38	123.3 (2)	C59—C61'—H61F	109.5
C37—C36—C38	120.8 (2)	H61D—C61'—H61F	109.5
C10—C37—C36	123.1 (2)	H61E—C61'—H61F	109.5
C10—C37—H37	118.4	C59—C62'—H62D	109.5
C36—C37—H37	118.4	C59—C62'—H62E	109.5
C41—C38—C40	109.3 (2)	H62D—C62'—H62E	109.5
C41—C38—C39	108.8 (2)	C59—C62'—H62F	109.5
C40—C38—C39	107.3 (2)	H62D—C62'—H62F	109.5
C41—C38—C36	110.1 (2)	H62E—C62'—H62F	109.5
C40—C38—C36	109.4 (2)		
			
C21—O1—C1—C2	88.0 (2)	C28—C29—C31—C33	1.2 (4)
C21—O1—C1—C20	−97.9 (2)	C30—C29—C31—C34	54.1 (5)
O1—C1—C2—C58	174.25 (18)	C28—C29—C31—C34	−124.9 (5)
C20—C1—C2—C58	0.2 (3)	C30—C29—C31—C32'	−128.4 (7)
O1—C1—C2—C3	0.1 (3)	C28—C29—C31—C32'	52.6 (8)
C20—C1—C2—C3	−173.9 (2)	C30—C29—C31—C32	−62.7 (4)
C1—C2—C3—C4	130.4 (2)	C28—C29—C31—C32	118.3 (4)
C58—C2—C3—C4	−43.7 (3)	C30—C29—C31—C33'	118.6 (6)
C2—C3—C4—C28	123.0 (2)	C28—C29—C31—C33'	−60.3 (7)
C2—C3—C4—C5	−54.0 (3)	C9—C8—C35—C36	−2.5 (3)
C28—C4—C5—O2	179.8 (2)	C7—C8—C35—C36	174.43 (19)
C3—C4—C5—O2	−3.1 (3)	C8—C35—C36—C37	−2.4 (3)
C28—C4—C5—C6	0.0 (3)	C8—C35—C36—C38	−178.8 (2)
C3—C4—C5—C6	177.1 (2)	C9—C10—C37—C36	0.6 (3)
O2—C5—C6—C30	−177.98 (18)	C11—C10—C37—C36	−176.65 (19)
C4—C5—C6—C30	1.8 (3)	C35—C36—C37—C10	3.4 (3)
O2—C5—C6—C7	5.7 (3)	C38—C36—C37—C10	179.9 (2)
C4—C5—C6—C7	−174.50 (18)	C35—C36—C38—C41	−135.0 (3)
C30—C6—C7—C8	−93.9 (2)	C37—C36—C38—C41	48.7 (3)
C5—C6—C7—C8	82.3 (2)	C35—C36—C38—C40	104.9 (3)
C6—C7—C8—C9	−126.2 (2)	C37—C36—C38—C40	−71.4 (3)
C6—C7—C8—C35	57.0 (3)	C35—C36—C38—C39	−13.9 (3)
C35—C8—C9—O3	−176.16 (18)	C37—C36—C38—C39	169.9 (2)
C7—C8—C9—O3	7.1 (3)	C13—C12—C42—C43	−2.8 (3)
C35—C8—C9—C10	6.7 (3)	C11—C12—C42—C43	173.2 (2)
C7—C8—C9—C10	−170.06 (18)	C12—C42—C43—C44	−1.2 (3)
C8—C9—C10—C37	−5.8 (3)	C12—C42—C43—C45	179.3 (2)
O3—C9—C10—C37	176.79 (18)	C42—C43—C44—C14	2.9 (3)
C8—C9—C10—C11	171.38 (18)	C45—C43—C44—C14	−177.67 (19)
O3—C9—C10—C11	−6.0 (3)	C13—C14—C44—C43	−0.3 (3)
C37—C10—C11—C12	−81.4 (3)	C15—C14—C44—C43	−179.39 (19)
C9—C10—C11—C12	101.4 (2)	C44—C43—C45—C47	108.0 (3)
C10—C11—C12—C13	−92.6 (3)	C42—C43—C45—C47	−72.5 (3)
C10—C11—C12—C42	91.6 (2)	C44—C43—C45—C48'	−74.4 (8)
C42—C12—C13—C14	5.5 (3)	C42—C43—C45—C48'	105.0 (8)
C11—C12—C13—C14	−170.40 (19)	C44—C43—C45—C47'	45.6 (9)
C42—C12—C13—O4	−177.51 (18)	C42—C43—C45—C47'	−134.9 (9)
C11—C12—C13—O4	6.6 (3)	C44—C43—C45—C48	−14.2 (4)
C12—C13—C14—C44	−4.0 (3)	C42—C43—C45—C48	165.3 (3)
O4—C13—C14—C44	178.87 (17)	C44—C43—C45—C46'	167.5 (9)
C12—C13—C14—C15	175.02 (19)	C42—C43—C45—C46'	−13.1 (9)
O4—C13—C14—C15	−2.1 (3)	C44—C43—C45—C46	−131.1 (3)
C13—C14—C15—C16	97.2 (2)	C42—C43—C45—C46	48.4 (3)
C44—C14—C15—C16	−83.8 (2)	C17—C16—C49—C50	−1.5 (3)
C14—C15—C16—C17	−89.7 (2)	C15—C16—C49—C50	−179.0 (2)
C14—C15—C16—C49	87.8 (2)	C16—C49—C50—C51	−0.8 (3)
C49—C16—C17—O5	−177.51 (18)	C16—C49—C50—C52	174.7 (2)
C15—C16—C17—O5	0.0 (3)	C17—C18—C51—C50	−0.7 (3)
C49—C16—C17—C18	2.7 (3)	C19—C18—C51—C50	170.31 (19)
C15—C16—C17—C18	−179.79 (19)	C49—C50—C51—C18	1.9 (3)
C16—C17—C18—C51	−1.7 (3)	C52—C50—C51—C18	−173.7 (2)
O5—C17—C18—C51	178.52 (18)	C49—C50—C52—C55	−105.1 (3)
C16—C17—C18—C19	−172.71 (18)	C51—C50—C52—C55	70.1 (3)
O5—C17—C18—C19	7.5 (3)	C49—C50—C52—C53'	70.9 (11)
C51—C18—C19—C20	−92.5 (2)	C51—C50—C52—C53'	−113.8 (11)
C17—C18—C19—C20	78.2 (2)	C49—C50—C52—C53	132.3 (3)
C2—C1—C20—C56	0.1 (3)	C51—C50—C52—C53	−52.4 (3)
O1—C1—C20—C56	−173.96 (17)	C49—C50—C52—C54'	−51.6 (11)
C2—C1—C20—C19	176.6 (2)	C51—C50—C52—C54'	123.6 (11)
O1—C1—C20—C19	2.6 (3)	C49—C50—C52—C55'	−168.6 (9)
C18—C19—C20—C56	14.7 (3)	C51—C50—C52—C55'	6.6 (9)
C18—C19—C20—C1	−161.70 (19)	C49—C50—C52—C54	15.0 (3)
C1—O1—C21—C22	−168.44 (19)	C51—C50—C52—C54	−169.7 (2)
O1—C21—C22—C27	−134.9 (3)	C1—C20—C56—C57	0.6 (3)
O1—C21—C22—C23	44.4 (3)	C19—C20—C56—C57	−175.9 (2)
C27—C22—C23—C24	−0.4 (4)	C20—C56—C57—C58	−1.5 (3)
C21—C22—C23—C24	−179.7 (2)	C20—C56—C57—C59	174.23 (19)
C22—C23—C24—C25	0.3 (4)	C56—C57—C58—C2	1.8 (3)
C23—C24—C25—C26	0.8 (5)	C59—C57—C58—C2	−173.8 (2)
C24—C25—C26—C27	−1.8 (6)	C1—C2—C58—C57	−1.3 (3)
C23—C22—C27—C26	−0.6 (5)	C3—C2—C58—C57	173.0 (2)
C21—C22—C27—C26	178.7 (3)	C58—C57—C59—C61'	−57.1 (10)
C25—C26—C27—C22	1.7 (5)	C56—C57—C59—C61'	127.4 (10)
C5—C4—C28—C29	−1.3 (3)	C58—C57—C59—C60	107.6 (4)
C3—C4—C28—C29	−178.4 (2)	C56—C57—C59—C60	−67.9 (4)
C4—C28—C29—C30	0.6 (3)	C58—C57—C59—C62	−130.8 (5)
C4—C28—C29—C31	179.6 (2)	C56—C57—C59—C62	53.7 (5)
C28—C29—C30—C6	1.3 (3)	C58—C57—C59—C60'	62.5 (11)
C31—C29—C30—C6	−177.7 (2)	C56—C57—C59—C60'	−113.0 (11)
C5—C6—C30—C29	−2.5 (3)	C58—C57—C59—C61	−10.1 (5)
C7—C6—C30—C29	173.72 (19)	C56—C57—C59—C61	174.5 (4)
C30—C29—C31—C34'	8.1 (7)	C58—C57—C59—C62'	−177.9 (9)
C28—C29—C31—C34'	−170.9 (6)	C56—C57—C59—C62'	6.6 (9)
C30—C29—C31—C33	−179.8 (4)		

### Hydrogen-bond geometry (Å, º)

**Table:**

*D*—H···*A*	*D*—H	H···*A*	*D*···*A*	*D*—H···*A*
O3—H3···O5^i^	0.82	2.04	2.7197 (19)	140
O4—H4···O3	0.82	2.04	2.847 (2)	168
O5—H5···O4	0.82	2.00	2.803 (2)	168

Symmetry code: (i) −*x*+1, −*y*, −*z*+1.
